# Enhanced Gamma Ray Radiation Resistance of Silicone Elastomers via Trace Addition of Perovskite Nanocrystals for Free Radicals Scavenging

**DOI:** 10.1002/smsc.202400470

**Published:** 2025-01-07

**Authors:** Wei Zheng, Xinyi Han, Jinghao Hao, Han Liu, Teng Long, Lin Zhu, Haifeng Lu, Hua Wang, William W. Yu, Chuanjian Zhou

**Affiliations:** ^1^ School of Materials Science and Engineering Shandong University Jinan 250061 China; ^2^ Key Laboratory of Special Functional Aggregated Materials Ministry of Education Jinan 250100 China; ^3^ School of Chemistry & Chemical Engineering Shandong University Jinan 250100 China; ^4^ Shandong Provincial Key Laboratory for Science of Material Creation and Energy Conversion Science Center for Material Creation and Energy Conversion Qingdao 266237 China; ^5^ School of Chemistry and Chemical Engineering, Shandong University, Jinan Shandong Key Laboratory of Advanced Organosilicon Materials and Technologies Jinan 250100 China

**Keywords:** free radicals, perovskite nanocrystals, resistance to irradiation, silicone elastomers

## Abstract

Halide perovskites exhibit remarkable properties, such as high optical absorption coefficient, excellent defect tolerance, simple and cheap preparation process, etc., especially excellent radiation hardness, which makes them used in high‐energy ray radiation environments. Free radicals, with singly occupied molecular orbitals, are highly reactive intermediates that play a critical role in material degradation under ray radiation. However, the interaction between these radicals and halide perovskites remains inadequately understood, despite its crucial for enhancing the γ‐ray resistance of elastomers. This study investigates the interfacial charge transfer between halide perovskite nanocrystals (PNCs) and silicon, carbon, and hydrogen radicals, supported by theoretical calculations and electron spin resonance analyses, revealing the immobilization of reactive radicals on the PNC surface. To further investigate, amino‐propyl triethoxysilane (APTES)‐passivated PNCs are synthesized and incorporated into silicone elastomers via an in situ one‐step crosslinking process. Under high‐dose γ‐ray irradiation, these elastomers generate free radicals that typically degrade the material. PNCs effectively stabilize these radicals, improving the elastomer's γ‐ray resistance, with only an 11% loss in mechanical strength after 300 KGy, one‐fifth of the loss in unmodified silicones elastomers. This study provides valuable insights for developing radiation‐resistant perovskite polymer composites for aerospace and nuclear industries.

## Graphical abstract

The addition of perovskite nanocrystals (PNCs) significantly mitigated free radical damage during γ‐ray irradiation, enhancing the irradiation resistance of silicone elastomers. After 300 kGy γ‐ray exposure, PNC‐silicone elastomers retained 89% mechanical strength, twice that of counterparts without PNCs (43%). Density functional theory calculations revealed that resonance structure is obtained by the interfacial charge transfer between the PNCs and radicals.

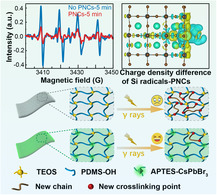

## 1 Introduction

The exceptional properties of halide perovskites, including their high optical absorption coefficient, remarkable defect tolerance, and outstanding radiation resistance, have garnered significant attention in the field of aerospace applications.^[^ ^1^, ^2^, ^3^, ^4^ ^]^ The perovskite solar cells (PSCs) were fabricated onto polymer films and subsequently integrated into a rocket payload, which was launched to an apogee of 239 km for conducting space flight experiments aimed at monitoring the current–voltage characteristics of the cells under controlled irradiation conditions.^[^ ^5^ ^]^ The impact of environmental stressors, such as moisture and oxygen, on the stability of perovskites is widely acknowledged; however, these stresses can be significantly mitigated in a space environment.^[^ ^6^ ^]^ Furthermore, Huang et al. reported that PSCs exhibit a remarkable stability, with 96.8% of their initial efficiency retained even after continuous irradiation at a cumulative γ‐ray dose of 2.3 Mrad. This exceptional stability can be attributed to the self‐healing behavior of perovskites, wherein the replaced ions spontaneously return to their original defect positions due to thermodynamic processes, indicating halide perovskites exhibit promising potential under high γ‐ray irradiation.^[^ ^7^ ^]^

Recently, polymers and small molecules possessing free radicals, which are characterized by the presence of unpaired electrons in their molecular structure, have been employed as passivators to improve the efficiency and stability of PSCs.^[^ ^8^ ^]^ Wang et al. introduced a redox‐active radical polymer, poly(oxoammonium salt), which enables P‐doping of the hole transport material through hole injection, thereby optimizing its Fermi energy level and conductivity and improving electrical property stability of the hole‐transporting layer.^[^ ^9^ ^]^ Xu's group designed a stabilized free radical derivative (OTTM) containing methoxy and polychlorinated radicals, in which the strong electron delocalization between the donor portion of the free radicals and the acceptor portion of the Cl group effectively improves PSCs crystallization and reduces defects in their layer.^[^ ^10^ ^]^ Gao et al. designed D‐A‐type nanographene molecules (D‐A NG‐tBu and D‐A NG‐OMe) with an electron‐deficient graphene core and an electron‐rich aniline derivative at the periphery. Electron spin resonance experiments revealed that this conjugated structure facilitates π‐electron delocalization, resulting in the formation of stable free radicals. The introduction of these graphene molecules at the interface between PSCs and the hole transport layer enabled regulation of carrier transport. Consequently, the power conversion efficiencies for devices modified with D‐A NG‐OMe and D‐A NG‐tBu reached 23.47% and 23.51%, respectively.^[^ ^11^ ^]^ The ortho‐squaraine derivative designed by Niu et al. features an inner carbonyl group serving as the electron‐donor unit and a benzene ring acting as the external unit. It possesses a unique single‐electron resonance structure in its ground state, influencing the charge state on the surface of PSCs and resulting in band bending that facilitates efficient charge extraction and transfer from the PSCs layer to the hole transport layer.^[^ ^12^ ^]^ In summary, several types of stable‐free radical molecules have been reported to modify PSCs to effectively improve their efficiency and stability of the devices. However, the interaction between highly reactive radicals and perovskite materials remains largely unexplored, while such highly reactive and unstable radicals exist in various fields including organic synthesis, material degradation, and photocatalysis.

The unique properties of silicone elastomers, such as their remarkable resistance to extreme temperatures, durability against weather conditions, and resilience to ozone exposure, have made them widely utilized in nuclear facility and deep space exploration where high‐energy rays irradiation environment prevail.^[^ ^13^, ^14^, ^15^ ^]^ The exposure of silicone elastomers to high‐energy radiation in space environments can result in significant degradation of their performance and lifespan due to cross‐linking or cleavage reactions.^[^ ^16^ ^]^ The main chain or side groups of polydimethylsiloxane (PDMS) undergo irradiation will generate highly reactive free radicals. These free radicals exhibit remarkable reactivity, leading to their attack on the main chain of PDMS and resulting in intricate crosslinking processes that give rise to a highly cross‐linked network.^[^ ^17^ ^]^ This leads to reduced chain segment mobility and increased brittleness, thereby significantly constraining the application potential of silicone elastomers in the nuclear facility and aerospace industry.^[^ ^18^ ^]^ A series of measures have been developed to improve the irradiation resistance of silicone elastomers. The irradiation resistance of silicone elastomers was studied by Todica et al. through the incorporation of titanium dioxide particles, resulting in obvious improvements in their mechanical properties after exposure to radiation.^[^ ^19^ ^]^ Zhao et al. incorporated yttrium phosphate at a mass fraction of 20% into silicone elastomers, resulting in a significantly improved irradiation resistance.^[^ ^20^ ^]^ The minimum mass fraction of 20% for current irradiation resistance additives is required for achieving a certain level of irradiation resistance.^[^ ^21^ ^]^ The mechanical properties of silicone elastomers, however, are somewhat influenced by the extensive presence of additives due to the limited compatibility between additives and silicone elastomers. Moreover, the cost of spacecraft significantly increases with each incremental unit weight. Therefore, it is urgent to develop a high‐efficiency radiation resistant additive for silicone elastomers, with only a minimal quantity required.

Here, we report the first study of the combination and interfacial charge transfer between CsPbBr_3_ PNCs and free radicals (including hydrogen, carbon, and silicon radical), which are essential processes for improving resistance against γ‐ray irradiation of silicone elastomer. CsPbBr_3_ PNCs with a high surface coverage of silica ethoxylates, employing poly(maleic anhydride‐alt‐1‐octadecene) (PMAO) and amino‐propyl triethoxysilane (APTES) as ligands, were chosen for this study due to their chemical stability and compatibility with silicone elastomer. Density functional theory (DFT) calculations explored the combination and interfacial charge transfer between CsPbBr_3_ PNCs and free radicals, leading to stable free radical complex intermediate. Electron spin resonance (ESR) and curing curves further indicated that PNCs efficiently trapped all kinds of free radicals and reduced the concentration of free radicals in system. Both pristine silicone elastomers and those containing PNCs were exposed to a cumulative dose of 300 KGy γ‐ray radiation over seven days (radiation dose equivalent to a 10‐year satellite in Earth orbit, ≈26 KGy year^−1^).^[^ ^22^ ^]^ The decay rate of the mechanical properties of PNCs‐incorporated silicone elastomers was effectively suppressed compared to that of no PNCs‐silicone elastomers. Dynamic mechanical analysis (DMA) and magnetic resonance crosslink density spectra confirmed that the presence of PNCs effectively suppressed the significant increase in crosslink density of silicone elastomers following irradiation. Consequently, PNCs‐enhanced silicone elastomers with exceptional resistance to irradiation offer a solid foundation for potential applications in aerospace and nuclear industries.

## 2 Results and Discussion

CsPbBr_3_ PNCs were synthesized using the ligand‐assisted reprecipitation method at room temperature, and subsequently modified with PMAO and APTES ligands. The synthesis experiments of APTES‐PNCs were successfully scaled up by tenfold and 20‐fold, while still exhibiting exceptional optical performance (Figure S1, Supporting Information), thereby indicating their potential for future practical applications. As a polymer multidentate ligand, PMAO effectively enhances the interaction with the PNCs surface, thereby improving their stability.^[^ ^23^, ^24^ ^]^

**Figure 1:**
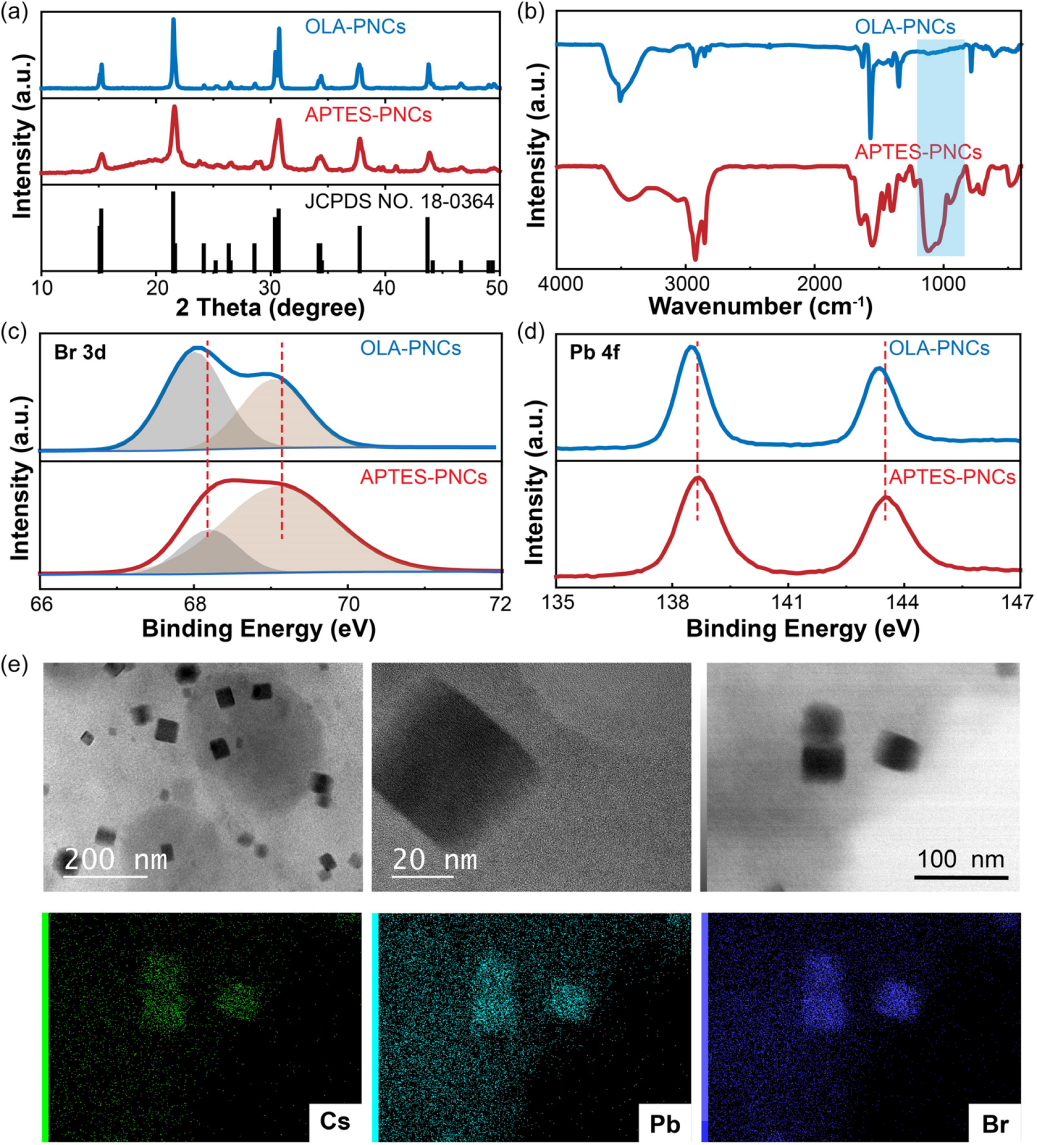
Structural characterization of CsPbBr_3_ PNCs. a) XRD patterns and b) FT‐IR spectra of OLA‐PNCs and APTES‐PNCs. c,d) XPS spectra of PNCs, the PNCs synthesized by APTES showed higher binding energy. e) TEM images and EDS elemental mappings of APTES‐PNCs, showing the perfect encapsulation of PNCs with APTES.

To investigate the impact of APTES modification on the structure of PNCs, we first characterized APTES‐PNCs in various physical phases. **Figure** **1**a illustrates the X‐ray diffraction (XRD) pattern of the PNCs associated with a cubic structure. Compared to oleylamine‐PNCs (OLA‐PNCs), slight shifts were observed in the XRD peaks of APTES‐PNCs, which could be attributed to lattice strain resulting from PMAO and APTES ligand adsorption.^[^ ^15^ ^]^ Furthermore, indexing analysis revealed that the XRD pattern corresponds to the cubic phase (Inorganic Crystal Structure Database (ICSD) no. 18–0364).^[^ ^25^ ^]^ Subsequently, Fourier transform infrared spectroscopy (FT‐IR) analysis was conducted, revealing the presence of N—H stretching vibrational absorption bands at 3420 cm^−1^ and C=O stretching vibrational absorption bands at 1720 cm^−1^ in the spectrum of OLA‐PNCs (Figure 1b). Meanwhile, distinct characteristic absorption bands of Si—O—Si were observed at 1050 and 960 cm^−1^ in the APTES‐PNCs spectrum, providing evidence for the formation of cross‐linked networks on the surfaces of PNCs through APTES.^[^ ^26^ ^]^ The X‐ray photoelectron spectroscopy (XPS) spectra of the samples were also analyzed to compare the interactions between different ligands and the surface of PNCs. In Figure S2, Supporting Information, major elements Cs, Pb, and Br in PNCs were detected along with surface ligand‐related elements C, N, Si, and O in the full spectrum. The binding energies of Br 3d and Pb 4f were found to be higher for APTES‐PNCs compared to OLA‐PNCs (Figure 1c,d). This finding indicates that the hydrolysis‐induced formation of a Si—O—Si network by APTES exhibits a stronger interaction with the surface of PNCs.^[^ ^27^ ^]^ The morphology and structure of APTES‐PNCs were characterized using transmission electron microscopy (TEM). As depicted in Figure 1e, the CsPbBr_3_ PNCs exhibited a predominantly cubic morphology, while the energy dispersive X‐ray spectroscopy (EDS) elemental maps clearly demonstrated the concentrated accumulation of Cs, Pb, and Br elements within these cubes, indicating the successful formation of CsPbBr_3_ PNCs.

**Figure 2:**
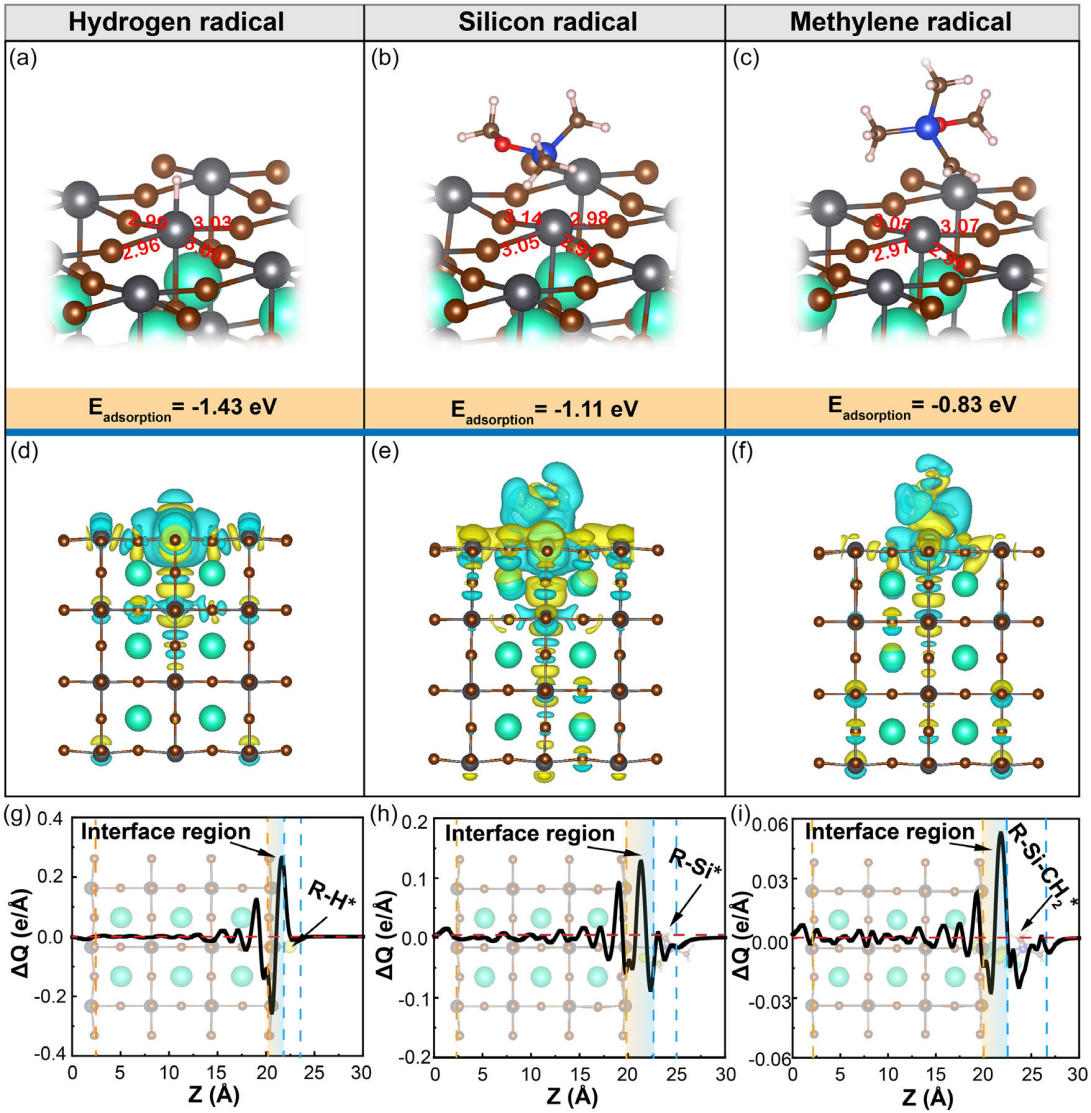
DFT calculation for interaction on PNCs surface with hydrogen radicals, silicon radicals, and methylene radicals. a–c) Optimized crystal structures for CsPbBr_3_ slab models with free radicals’ interaction. Chemical unit of bond length was Å. d–f) Charge density difference profiles of CsPbBr_3_ slab models with free radicals’ interaction. Blue and yellow represent electron depletion and electron accumulation with the isosurface value of 6e^−005^ e/Bohr^3^, respectively. g–i) CDC of PNCs/free radicals’ interfaces along the *Z* direction.

The interaction mechanism between highly reactive radicals and PNCs remains elusive, despite their wide presence in the fields of material degradation, photocatalysis, and organic synthesis. Here, the interaction mechanisms of hydrogen radicals, silicon radicals, and methylene radicals were explored by DFT calculations. The adsorption energies of the free radicals with Pb and Br atoms in the PNCs were both calculated. As presented in Table S1, Supporting Information, these free radicals exhibited high adsorption energies with the PNCs. Notably, the adsorption energy of free radicals with Pb atoms was consistently higher than that of Br atoms, suggesting a greater propensity for binding between the free radicals and Pb atoms. As shown in **Figure** **2**a–c, the adsorption energy of the three free radicals after binding with Pb atoms was −1.43, −1.11, and −0.83 eV, respectively, providing the robust interactions between PNCs and the free radicals.^[^ ^28^, ^29^ ^]^ The pristine PNCs exhibited a Pb—Br bond length of 3.02 Å, whereas the optimized structural model revealed significant variations in the Pb—Br bond lengths upon adsorption of free radicals (Table S2, S3, Supporting Information). This observation suggests pronounced lattice distortion in the PNCs upon interaction with free radicals. Subsequently, to further explore the charge transfer mechanism of free radicals upon adsorption by PNCs, the charge density difference and the Bader charge were calculated. The charge density difference equation as:

(1) $$\Delta \rho =\rho_{\text{PNCs}+\text{free radicals}}-\rho_{\text{PNCs}}-\rho_{\text{free radicals}}$$

$\rho_{\text{PNCs}+\text{free radicals}}$, $\rho_{\text{PNCs}}$, and $\rho_{\text{free radicals}}$ are the charge densities of PNCs/free radicals slab models, and the corresponding isolated PNCs and free radicals, respectively. As shown in Figure 2d–f, charge depletion (blue region) and charge accumulation (yellow region) mainly occurred in the interfacial region between the free radicals and PNCs, and the alternating charge accumulation and depletion layers made the interface reached a more stable state, indicating the powerful interaction between them.^[^ ^30^ ^]^ The free radicals generated upon irradiation of silicone elastomers generally have unique pairs of electrons and high activity. Bader charge calculations were performed to elucidate the extent of electron transfer following the entrapment of highly reactive radicals by PNCs. The results revealed that the hydrogen radical got 0.244e and the silicon and methylene radicals lost 0.2121 and 0.0062e, respectively (Table S4, Supporting Information). Consequently, various free radicals experienced diverse degrees of electron gain and loss upon being trapped by PNCs. Effective charge transfer led to a reduction in the activity of free radicals, promoting their transition toward more stable states.^[^ ^31^ ^]^

The charge displacement curves (CDCs) were analyzed by integrating the charge density difference along the *Z*‐direction, for a more intuitive view of the charge transport between free radicals/PNCs. The equation as:

(2) $$\Delta Q=\int_{-\infty }^{\infty }dx\int_{-\infty }^{\infty }dy\int_{-\infty }^{z}\Delta \rho dz$$

According to this equation, a positive Δ*Q* means electrons transferring from right to left across the normal plane.^[^ ^32^ ^]^ It was noted that the positive (negative) slopes of the CDC represent the charge accumulation (depletion) in the corresponding regions. As shown in Figure 2g–i, the CDC for different free radicals/PNCs was demonstrated. In the interface region, higher peaks of electron number were observed compared to the region of PNCs and free radicals, indicating a significant charge transfer following adsorption. This observation further proved the strong interface coupling and significant charge transfer process between the radicals and PNCs, thus the radical molecules and PNCs form a stable resonance structure.^[^ ^33^ ^]^

**Figure 3:**
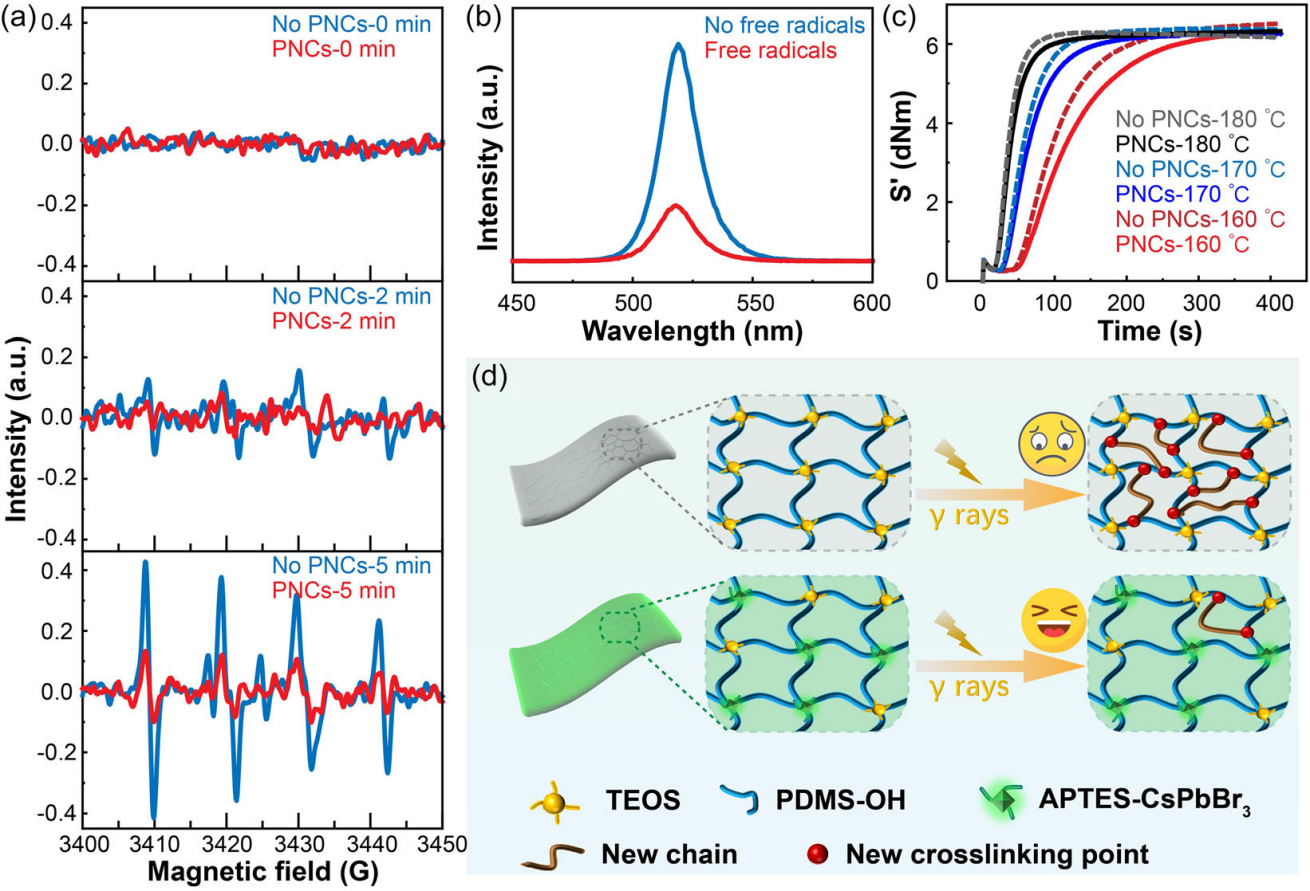
The study on the irradiation resistance mechanism of PNCs‐silicone elastomers. a) ESR spectra of the photoinitiator were obtained in the presence and absence of PNCs. The ESR data were collected at 0, 2, and 5 min of irradiation under a 365 nm UV lamp, respectively. b) PL spectra of APTES‐PNCs with and without free radicals. c) The curing curves of the initiator at temperatures of 160, 170, and 180 °C. d) Schematic diagram illustrating the mechanism by which PNCs enhance irradiation resistance in silicone elastomers.

Multiple experiments were further conducted to observe the influence of PNCs on free radicals in the system. Trimethylbenzoyl‐diphenylphosphine oxide (TPO), a photoinitiator, undergoes decomposition upon exposure to 365 nm UV light, resulting in the generation of numerous free radicals. The ESR spectrum can be utilized for the detection of these generated free radicals, with signal intensity being positively correlated to their concentration. Consequently, ESR spectra were measured in both the photoinitiator system with and without the addition of PNCs (see supporting information for details). **Figure** **3**a illustrates the characterization of ESR spectra for TPO solution and mixed TPO + PNCs solution at 0, 2, and 5 min under 365 nm UV illumination. Notably, no signal was observed at 0 min indicating an absence of free radical production. Subsequently, upon exposure to UV light for 2 min, the TPO solution exhibited an evident ESR signal, indicative of the generation of free radicals through decomposition. Following a 5‐min irradiation period, the signal intensified significantly, suggesting a higher production of free radicals via decomposition. In comparison to the TPO solution alone, both at 2 and 5 min of UV irradiation, there was a notable reduction in the ESR signal observed in the mixed solution containing TPO + PNCs. This observation implies that the addition of PNCs effectively diminishes the concentration of free radicals within this system. Consequently, it can be concluded that PNCs possess efficient scavenging capabilities toward free radicals in this context.^[^ ^34^ ^]^

The photoluminescence (PL) spectra of the PNCs system, both in the presence and absence of free radicals, were compared to further elucidate the interaction between PNCs and free radicals. TPO and PNCs were mixed in a toluene solution and divided into two equal groups. In one group, TPO and PNCs were exposed to a 365 nm UV lamp for 2 min to generate free radicals, followed by measurement of PL spectra (with free radicals). The other group was directly measured for PL spectra (no free radicals) (see supporting information for details). A comparison of the PL spectra from these two groups clearly indicated that the luminescence intensity of PNCs significantly decreased when a substantial number of free radicals were generated within the system (Figure 3b). The decrease in luminescence can be attributed to lattice distortion in PNCs induced by the combination of free radicals, which is consistent with the theoretical calculation, resulting in an enhanced non‐radiative recombination. Furthermore, to exclude the influence of UV light, we conducted experiments investigating the interaction between PNCs and free radicals in the absence of UV irradiation. Peroxide initiator 2,5‐dimethyl‐2,5‐bis(tert‐butylperoxy) hexane (DBPH) was employed to generate a substantial amount of free radicals at elevated temperatures. Upon mixing DBPH with polydimethylsiloxane raw rubber (PDMS‐2.4%Vi), these generated free radicals reacted with the vinyl groups present in PDMS, resulting in the formation of three‐dimensional crosslinked networks and facilitating the preparation of high‐temperature vulcanized silicone elastomer. The curing curves of PDMS with and without added PNCs were measured at temperatures of 160, 170, and 180 °C, respectively. As depicted in Figure 3c, the rate of initiator decomposition increased with temperature, leading to a higher production rate of free radicals, and consequently, accelerating the PDMS curing process. However, when PNCs were incorporated into PDMS under identical conditions, a significant reduction in the curing rate was observed. At lower heating temperatures, the initiator was decomposed at a lower rate, consequently the scavenging effect of PNCs on free radicals was more obvious in comparison. These findings demonstrate that PNCs effectively retard the vulcanization rate of PDMS‐2.4%Vi by timely scavenging the generated free radicals, thereby enhancing the irradiation resistance of silicone elastomers.^[^ ^35^ ^]^ Through multiple experiments, we have clearly observed that PNCs effectively reduced the concentration of free radicals in the system. Unfortunately, lots of harmful and highly reactive free radicals are produced in some scenarios. This dilemma can be well solved by using the free radical scavenging effect of PNCs.

Representatively, silicone elastomers generate numerous highly reactive free radicals when subjected to high‐energy radiation. These free radicals can substantially degrade the mechanical properties of the polymers by damaging their crosslinked networks. When γ‐ray irradiation was applied to silicone elastomers without added PNCs, the PDMS main chain or side groups experienced breakage, resulting in the generation of a significant number of free radicals that subsequently underwent recombination reactions. The formation of Si—C—C—Si structure from two undercoordinated methyl side groups (Si—CH_2_*) led to the release of two H atoms and the production of H_2_. Similarly, Si—C—Si formation resulted in the release of an H atom and a CH_3_ group, leading to CH_4_ production. Additionally, direct Si—Si connections could be formed due to the loss of two CH_3_ side groups, simultaneously generating C_2_H_6_. Consequently, severe degradation occurred in the mechanical properties of silicone elastomers (Figure S3, Supporting Information). Upon γ‐ray irradiation, a substantial number of free radicals were generated within the PNCs‐silicone elastomers matrix, facilitating their interaction with each other. The unpaired electrons of these free radicals may engage in interactions with electrons or atoms present on the perovskite surface. Consequently, neutralization or elimination of the free radicals occurs, leading to their transformation into a stable state. This process significantly reduces their reactivity and effectively inhibits the degradation of the crosslinked network in silicone elastomers caused by these highly reactive species (Figure 3d).^[^ ^18^, ^36^ ^]^ The addition of PNCs to silicone elastomers effectively enhances their resistance to irradiation. We propose hypotheses regarding the mechanism by which PNCs enhance the irradiation resistance of silicone elastomers: irradiation with γ‐rays generates numerous free radicals in the main chain or side groups of silicone elastomers, leading to destruction of their crosslinked network. The PNCs effectively scavenge these free radicals and eliminate them from the system, thereby inhibiting further damage to silicone elastomers caused by free radicals and enhancing their resistance to irradiation.

PNCs were introduced into silicone elastomers to investigate the effect of PNCs on improving the irradiation resistance of silicone elastomers. First, silicone elastomers were designed and synthesized based on PDMS‐OH, tetraethyl orthosilicate (TEOS), silica, and dibutyltin dilaurate (DBTL) under ambient conditions. The Si—O—Si crosslink network of the silicone elastomer was formed via the reaction between TEOS and PDMS‐OH. Cyclic tensile tests were conducted to investigate the material's elasticity and resistance to fatigue damage by stretching it to twice of its original length for ten cycles. In the initial cycle, significant hysteresis and irrecoverable deformation were observed; however, in subsequent cycles, the hysteresis loop remained nearly unchanged, indicating excellent elasticity and resistance to fatigue damage of the silicone elastomers (Figure S4, Supporting Information). Additionally, these silicone elastomers exhibited exceptional transparency, flexibility, and mechanical strength.

**Figure 4:**
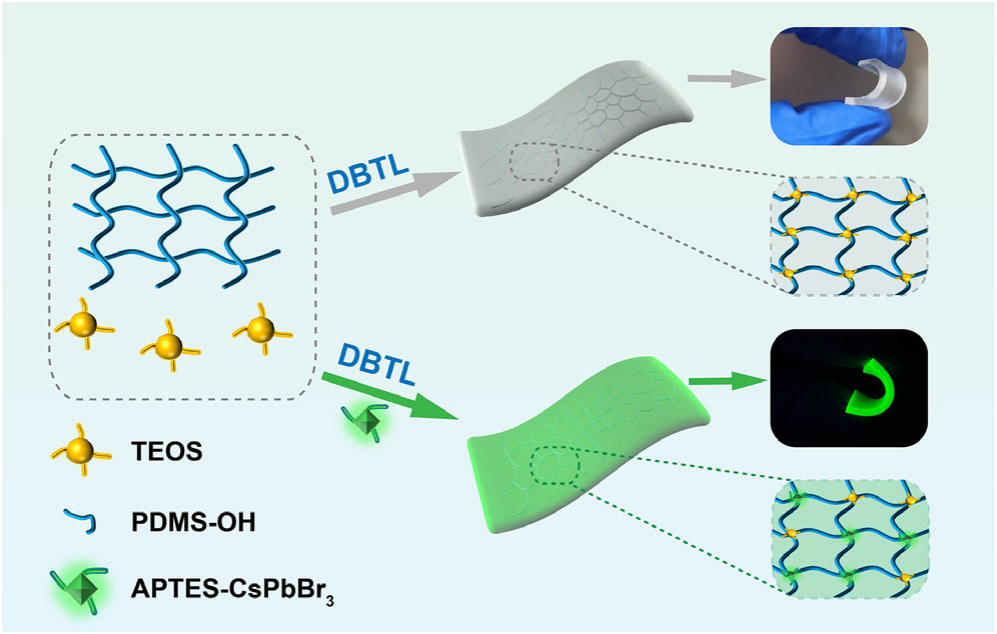
Schematic illustration of PNCs‐silicone elastomers prepared by room temperature curing.

The PNCs were further passivated by APTES ligands containing a high concentration of silicon ethoxy groups, which served as cross‐linking agents integrated into the Si—O—Si cross‐linking network upon blending with silicone elastomers. Consequently, PNCs‐silicone elastomers with exceptional fluorescent properties were successfully obtained, and the PNCs were more uniformly dispersed within the silicone elastomer through a chemically reactive process (**Figure** **4**), effectively addressing the compatibility issue between PNCs and silicon elastomer.

**Figure 5:**
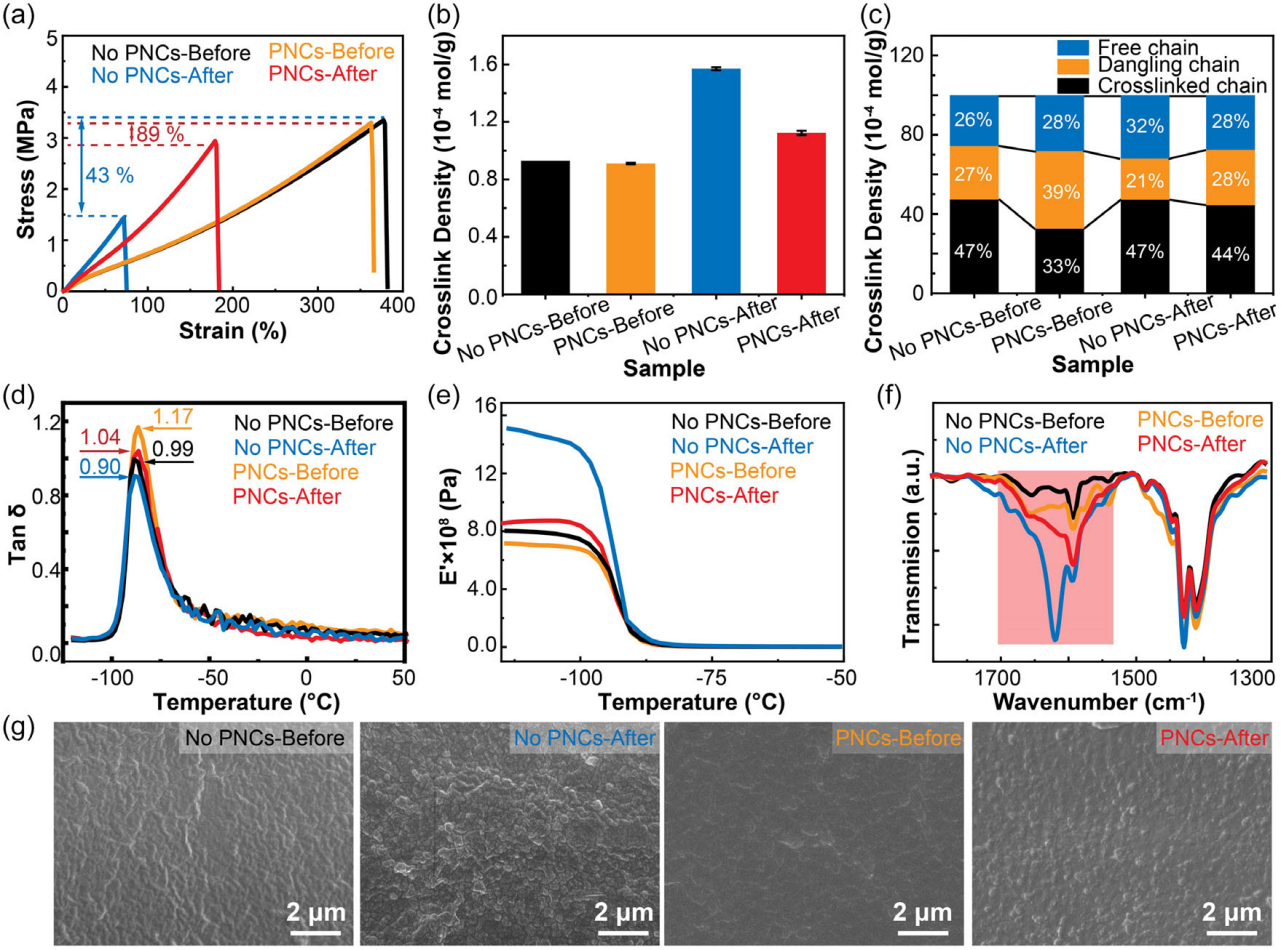
Characterization of irradiation properties of silicone elastomers (No PNCs) and PNCs‐silicone elastomers (PNCs). a) Stress–strain curves of silicone elastomers and PNCs‐silicone elastomers before and after irradiation. b) The cross‐linking density, as well as c) the specific gravity of crosslinked chain, dangling chain, and free chain in both silicone elastomers and PNCs‐silicone elastomers are presented before and after irradiation. d) Loss factor and e) storage modulus of DMA for silicone elastomers and PNCs‐silicone elastomers pre‐ and post‐irradiation. f) FT‐IR spectra and g) SEM of both silicone elastomer samples with or without PNCs before and after irradiation.

The simultaneous irradiation of both silicone elastomers and PNCs‐silicone elastomers with 300 KGy of γ‐rays was conducted to investigate the influence of PNCs on the radiation resistance of silicone elastomers. Remarkably, silicone elastomers containing 2.75% PNCs exhibited superior irradiation resistance (Table S5, Supporting Information). Subsequently, a comprehensive characterization was conducted to evaluate various property changes in both pristine silicone elastomers and PNCs‐silicone elastomers before and after γ‐ray irradiation. Initially, mechanical properties were evaluated through analysis of stress–strain curves. As depicted in **Figure** **5**a, fracture stresses for pristine silicone elastomers‐before γ‐ray irradiation (No PNCs‐Before) and PNCs‐silicone elastomers‐before γ‐ray irradiation (PNCs‐Before) were measured as 3.35 and 3.29 MPa, respectively, with corresponding stretch abilities of 381% and 366% prior to irradiation. Following exposure to a dose of 300 K Gy γ‐rays, the mechanical properties of silicone elastomers‐after γ‐ray irradiation (No PNCs‐After) deteriorated significantly, resulting in fracture stress values as low as 1.43 MPa and a reduction in stretch ability to ≈75%. In contrast, PNCs‐silicone elastomers‐after γ‐ray irradiation (PNCs‐After) exhibited excellent performance after irradiation with fracture stress values measuring at 2.94 MPa accompanied by a stretch ability retention rate reaching up to ≈184%. This clearly demonstrates that the addition of PNCs effectively improves the irradiation resistance of silicone elastomers, leading to excellent mechanical properties even after exposure to a high‐dose γ‐ray irradiation.

To deeply understand the impact of PNCs on the crosslink density of silicone elastomers, magnetic resonance crosslink density spectra were analyzed (Figure 5b). Due to the low content of PNCs, the crosslink density of PNCs‐silicone elastomers (PNCs‐Before) before γ‐ray irradiation (0.91 × 10^−4^ mol g^−1^) did not significantly differ from that of silicone elastomers without PNCs before γ‐ray irradiation (No PNCs‐Before) (0.93 × 10^−4^ mol g^−1^). However, following γ‐ray irradiation, there was a notable increase in both crosslink density (from 0.93 × 10^−4^ to 1.57 × 10^−4^ mol g^−1^) and Shore hardness A (from 32 to 53) for silicone elastomers. It could be attributed to that the γ‐ray irradiation induced a secondary cross‐linking in the internal network of silicone elastomers, resulting in a significant increase in the crosslinking density. Consequently, the originally highly flexible silicone elastomer exhibited enhanced hardness and brittleness, which primarily contributed to the observed decline in mechanical properties.^[^ ^37^ ^]^ The crosslink density of PNCs‐silicone elastomers increased only from 0.91 × 10^−4^ to 1.12 × 10^−4^ mol g^−1^ after γ‐ray irradiation, while the Shore hardness A only exhibited a modest increase from 34 to 45 (Figure S5, Supporting Information). These findings indicate that the crosslinked network of silicone elastomers was effectively protected against excessive crosslinking induced by γ‐rays through the incorporation of PNCs, thereby preserving most of the original crosslinked network.

The specific gravity of free, crosslinked, and dangling chains in silicone elastomers and PNCs‐silicone elastomers was analyzed to gain a comprehensive understanding of the variations in cross‐linked density (Figure 5c). When silicone elastomers were irradiated with γ‐rays, the specific gravity of crosslinked chains remained unchanged, while the specific gravity of free chains was increased and that of dangling chains was decreased. This phenomenon can be attributed to the facile breakage of the dangling chains upon irradiation, resulting in the generation of additional free chains.^[^ ^38^ ^]^ In contrast, the specific gravity of the free chain in PNCs‐silicone elastomers remained unchanged after irradiation; the specific gravity of the dangling chain was decreased and that of the crosslinked chain was increased. It was possible that the PNCs were added to effectively reduce the occurrence of chain breaking and increased the participation of the polymer chains in the cross‐linking reaction, thus leading to the formation of more stable crosslinking chains in the PNCs‐silicone elastomers.^[^ ^39^ ^]^ It was postulated that the primary radicals were quickly scavenged by the PNCs during irradiation, and effectively inhibited the cross‐linking network from being further damaged, thus contributing to the stability of the silicone elastomers under irradiation conditions.^[^ ^40^ ^]^

Moreover, the effect of PNCs on the irradiation resistance of silicone elastomers was further explored by DMA characterization. As depicted in Figure 5d, the damping factor (tan δ) values of silicone elastomers were increased from 0.99 to 1.17 after adding PNCs. The elastomers with higher tan δ values usually have better irradiation resistance.^[^ ^41^ ^]^ The tan δ values of silicone elastomers were decreased from 0.99 to 0.9 after irradiation by γ‐rays, while the tan δ values of PNCs‐silicone elastomers were only decreased from 1.17 to 1.04. Notably, the tan δ values of PNCs‐silicone elastomers after irradiation were even significantly higher than those of silicone elastomers before irradiation. Since the free radicals generated during irradiation were promptly scavenged by the PNCs, further damage to the cross‐linked network was inhibited. This observation suggests that highly damped elastomers were able to better maintain their structure stability after being irradiated.^[^ ^42^ ^]^ The energy storage modulus and loss modulus of silicone elastomers exhibited a significant increase after irradiation by γ‐rays compared to PNCs‐silicone elastomers (Figure 5e, S6, Supporting Information). Silicone elastomers were cross‐linked twice, and the cross‐linking density was significantly increased due to the large number of free radicals generated during the irradiation process. The mobility of the molecular chains was significantly restricted due to the increased crosslink density.^[^ ^37^ ^]^

The effects of γ‐rays on the structure of silicone elastomers were characterized using FT‐IR spectra (Figure S7, Supporting Information). After γ‐ray irradiation, a prominent additional absorption peak at 1620 cm^−1^ appeared in the silicone elastomers, primarily attributed to C=O (Figure 5f). This observation suggests that Si—CH_3_ bonds within the crosslinked network were broken and C radicals reacted with oxygen molecules to form C=O in the surrounding environment. In contrast, no additional absorption peaks were observed in the IR spectra of PNCs‐silicone elastomers, providing compelling evidence for the effective protection offered by PNCs against γ‐ray damage.^[^ ^43^ ^]^ Scanning electron microscopy (SEM) characterization was conducted to visually compare the impact of γ‐rays on the morphology of silicone elastomers and PNCs‐silicone elastomers. It was evident that in the absence of PNCs, the surface of silicone elastomers experienced significant radiation degradation upon irradiation. Conversely, the irradiated surface of PNCs‐silicone elastomers remained intact as a continuous phase, indicating that PNCs effectively mitigated the destructive effects of γ‐rays on silicone elastomers (Figure 5g). This comprehensive set of characterizations substantiated that incorporating PNCs as cross‐linking agents significantly enhanced the radiation resistance of silicone elastomers. Based on this, we have demonstrated the effective limitation of free radical activities and reduction in their levels through both theoretical calculations and experimental characterization: The silicone elastomer was exposed to γ‐rays, resulting in the generation of numerous highly active free radicals within the system. Subsequently, charge transfer via PNCs effectively mitigated the reactivity of these free radicals. This intervention led to a significant reduction in free radical concentration and a notable decrease in reaction rate, as confirmed by ESR characterization and vulcanization curves. The inhibitory effect of PNCs effectively mitigated the detrimental impact of free radicals on the cross‐linked network of silicone elastomers, thereby significantly enhancing their resistance to irradiation. Consequently, PNCs‐silicone elastomers exhibit immense potential for application in the nuclear industry and deep space exploration.

## 3 Conclusion

In conclusion, the presence of effective interfacial charge transfer between PNCs and free radicals has been demonstrated through ESR characterization and DFT calculations, resulting in the stabilization of resonance structures and a substantial decrease in the concentration of free radicals within the system. The silicone elastomers were chemically cross‐linked with a small quantity of APTES‐PNCs incorporated, resulting in enhanced irradiation resistance by effectively inhibiting the destructive effect of free radicals generated in the high‐energy ray environment (300 KGy) on the elastomer network. After exposure to high doses of γ‐ray radiation, the mechanical strength reduction of PNCs‐silicone elastomers was only 11% of the initial value, in contrast to a substantial decrease of 57% observed for silicone elastomers without PNCs. The presence of PNCs was found to effectively suppress the significant increase in crosslink density of silicone elastomers after irradiation, as confirmed by DMA and magnetic resonance crosslink density spectra. Consequently, PNCs‐silicone elastomers exhibit promising application prospects in space exploration and the nuclear industry due to their exceptional resistance to irradiation.

## Conflict of Interest

The authors declare no conflict of interest.

## Author Contributions

**Wei Zheng**: conceptualization (lead); formal analysis (lead); methodology (lead); writing—original draft (lead). **Xinyi Han**: investigation (equal); methodology (equal). **Jinghao Hao**: investigation (equal); resources (equal). **Han Liu**: data curation (equal); validation (equal). **Teng Long**: software (lead); supervision (equal). **Lin Zhu**: supervision (equal); validation (supporting). **Haifeng Lu**: resources (equal); supervision (equal). **Hua Wang**: project administration (lead); resources (equal); writing—review & editing (lead). **William W. Yu**: supervision (equal); validation (equal); writing—review & editing (equal). **Chuanjian Zhou**: project administration (lead); writing—review & editing (lead).

## Supporting information

Supplementary Material

## Data Availability

The data that support the findings of this study are available from the corresponding author upon reasonable request.
