# Supplementary material for: Enhanced Gamma Ray Radiation Resistance of Silicone Elastomers via Trace Addition of Perovskite Nanocrystals for Free Radicals Scavenging
Source: Small Sci. 2025 Jan 7;5(4):2400470. doi: 10.1002/smsc.202400470 (PMC12244507; doi:10.1002/smsc.202400470)
Supplement: Supplementary file 1 — Supplementary Material [file SMSC-5-2400470-s001.pdf]

Supporting Information

Enhanced Gamma-Ray Radiation Resistance of Silicone Elastomers via Trace Addition of Perovskite Nanocrystals for Free Radicals Scavenging

Wei Zheng,[1] Xinyi Han,[1] Jinghao Hao,[1] Han Liu,[1] Teng Long,[1] Lin Zhu,[1] Haifeng Lu[2,3] Hua Wang,[1,2]* William W. Yu[2,3] and Chuanjian Zhou[1,2]**

[1]School of Materials Science and Engineering, Shandong University, Jinan, China

[2] Key Laboratory of Special Functional Aggregated Materials, Ministry of Education, Jinan, China

[3] School of Chemistry & Chemical Engineering, Shandong University, Jinan 250100; Shandong Provincial Key Laboratory for Science of Material Creation and Energy Conversion, Science Center for Material Creation and Energy Conversion, Qingdao 266237; Key Laboratory of Special Functional Aggregated Materials, Ministry of Education; Shandong Key Laboratory of Advanced Organosilicon Materials and Technologies, Jinan 250100, China

AUTHOR INFORMATION

Corresponding Authors

tenglong@sdu.edu.cn (Teng Long)

hwang@sdu.edu.cn (Hua Wang)

zhouchuanjian@sdu.edu.cn (Chuanjian Zhou)

Experimental section

Chemicals:

N,N-Dimethylformamide (DMF, 99.8%) and oleic acid (OA, 90%) was purchased from Sigma-Aldrich. Lead bromide ($PbBr_2$, 99.99%) and cesium bromide (CsBr, 99.99%) were purchased from Xian Polymer Light Technology Co., Ltd., China. Tetraethyl orthosilicate (TEOS, 99.99%), (3-aminopropyl) triethoxysilane (APTES, 99%), dibutyltin dilaurate (95%), oleylamine (95%) (OLA, 80-90%), 1-octadecene (ODE, 95%), dibenzoyl peroxide (BPO, 99.0%) and maleic anhydride (MA, 99.5%) were purchased from Shanghai Maclean Biochemical Technology Co., Ltd., China. SiO_2 nanoparticles (H2000) were purchased from Wacker Chemie AG, Germany. Hydroxyl-terminated polydimethylsiloxane (PDMS-OH, 10% Ph) was purchased from Shandong Dayi Chemical co., Ltd. Toluene and hexane were purchased from Sinopharm Chemical Reagent Co., Ltd. All chemicals were used as received without further purification.

Synthesis of Poly(maleic anhydride-alt-1-octadecene) (PMAO)

Briefly, 9.8 g of MA, 25 g of ODE and 36 g of toluene were added to a 250 mL three-neck flask. The mixture was dissolved by stirring under N_2 and room temperature for 60 min. Then, 2 g of BPO was added to the three-necked flask and the reaction was carried out at 105 °C for 8.5 h. After the reaction was completed, the solvent was removed by rotary evaporation. The obtained concentrate was washed with ethanol to remove impurities. Finally, the white powder was dried in a vacuum oven at 50 °C. It was stored in a glass vial for further use.

Synthesis of OLA-PNCs and APTES-PNCs

Briefly, 0.4 mmol of $PbBr_2$, 0.4 mmol of CsBr, 0.5 mL OA and 0.5 mL OLA were added to 10 mL of DMF solvent, and the precursor solution was obtained by sonication and vigorous stirring. Then 1 mL of the precursor solution was rapidly injected into 10 mL of toluene solution with vigorous stirring, which immediately transformed into bright green solution. Perovskite nanocrystal precipitates were obtained by centrifugation at 6000 rpm for 10 min and redispersed in 5 mL of hexane for further use.

The synthesis of APTES-PNCs was performed with essentially the same steps as for OLA-PNCs. The modification made was 0.5 mL OA and 0.5 mL OLA ligand was replaced with 0.2 g PMAO and 0.6 mL APTES ligand.

Chemical formulas and reaction schemes for synthesizing PNCs

The chemical synthesis formula for PNCs was as follows:

$$CsBr+PbBr_2 \rightarrow CsPbBr_3$$

The PNCs described in this manuscript were synthesized using a room-temperature ligand-assisted reprecipitation (LARP) method. The reaction procedure involved dissolving CsBr and $PbBr_2$ in *N*, *N*-dimethylformamide to prepare a precursor solution via ion exchange. The precursor solution was then rapidly injected into toluene, leading to the recrystallization of precursors and the formation of $CsPbBr_3$ PNCs. Ligands such as PMAO and APTES coordinated with the metal ions on the surface of the $CsPbBr_3$ PNCs, effectively passivating surface defects and preventing excessive growth of the nanocrystals

Preparation of Dehydrogenated Silicone Elastomers and PNCs-silicone Elastomers by Room Temperature Vulcanization

First, 30 g of hydroxyl-capped polydimethylsiloxane, 9 g of filler (gas phase silica), 1.2 g of cross-linking agent (tetraethyl orthosilicate) and 0.3 g of catalyst (dibutyltin dilaurate) were taken in the material container. They were mixed under 2000 rpm continuously for 60 s until all the raw materials were completely mixed well. Then it was poured into a polytetrafluoroethylene mold with a thickness of 2 mm and cured naturally at room condition for 24 h. After that, the successfully prepared silicone elastomers were slowly picked up from the mold for further measurement.

The synthesis of PNCs-silicone elastomers was performed with essentially the same steps as for silicone elastomers. The modification made was the addition of different quality fractions of APTES-PNCs while mixing the raw materials.

PL Spectral Measurement of APTES-PNCs System with and without Free Radicals

First, 2 mL of trimethylbenzoyl-diphenylphosphine oxide (TPO)-toluene solution (0.003 mmol/mL) was prepared, and then 0.5 mL of TPO-toluene solution was added to two groups of PNCs-toluene solution with the same concentration, respectively. One group of samples

was first irradiated by a 365 nm UV lamp for 2 min, which allowed TPO to produce many free radicals, and then the PL spectrum (with free radicals) was measured, and the other group of samples was directly measured PL spectrum (no free radicals). Then the luminous intensity of PNCs in different systems was compared.

Curing Curve Measurement

2,5-Dimethyl-2,5-bis(tert-butylperoxy) hexane (DBPH) acts as a peroxide initiator and generates many free radicals under high temperature conditions. After DBPH and polydimethylsiloxane (2.4% vinyl) (PDMS) were mixed uniformly, the resulting free radicals reacted with the vinyl groups in the PDMS in a curing reaction, forming a three-dimensional crosslinked network, and preparing high temperature cured silicone elastomers with excellent performance. Therefore, we prepared PDMS with and without PNCs, and measured the curing curves at temperatures of 160°C, 170°C and 180°C to observe the curing rate of PDMS under different conditions. The curing curves were obtained by rotorless vulcanometer (Mina-MDR, Prescott).

Transmission Electron Microscopic (TEM) Measurement

TEM images were obtained using a JEOL-JEM-2100 transmission electron microscope under an accelerating voltage of 200 kV.

X-ray Diffraction Measurement

X-ray diffraction (XRD) patterns of the synthesized perovskite nanocrystals were collected using an X-ray diffractometer (D/MAX-RC, Rigaku Corporation). The scanning rate was set at 10 °/min with a step size of 0.02°.

Scanning Electron Microscopy Imaging

The SEM images were collected by a scanning electron microscope (JSM-7610F, JEOL Ltd) under an acceleration voltage of 5 kV.

Steady-State Measurements of Photoluminescence

The photoluminescence (PL) spectra of PNCs colloid in toluene was collected by fluorescence spectrometer (FLS 1000, Edinburgh Instruments). The excitation wavelength was set at 365 nm, and PL was recorded with step size of 1 nm and dwell time of 0.1 s.

Fourier Transform Infrared (FT-IR) Spectra

The Fourier Transform Infrared (FT-IR) spectra of SE were characterized by a Fourier spectrometer (Tensor-37, Bruker). The scanning range was 4000-400 cm^{-1} and the step size was 1.93 cm^{-1}.

Mechanical Performance Measurement

The tensile strength and elongation at break of SE and PNCs-SE were characterized by an electronic universal testing machine (UTM-4203X, Shenzhen Sansi Zongheng Technology Co., Ltd.). The samples were measured using dumbbell-shaped sample strips according to GB/T-528-2009, and the loading speed was 500 mm/min.

Dynamic Mechanical Analysis (DMA) Measurement

The DMA spectra of SE and PNCs-SE were characterized by DMA instrumentation (ELF3220, BOSE). The measurement conditions were dynamic amplitude of 0.1 mm, a testing temperature interval of -130-50 °C, a testing frequency of 10 Hz, and a temperature increase rate of 6 °C/min.

Crosslink Density Measurement

The cross-linking density of SE and PNCs-SE was collected by NMR cross-linking density tester (VTMR20-010V-T, Suzhou Newmax Technology Co., Ltd.). The XLD2 fitting method was used, the measuring temperature was 35°C, and the average value was obtained by repeating three times for each sample.

Electron Spin Resonance (ESR) Measurement

The free radical concentration of the photoinitiators was characterized by electron spin resonance spectroscopy, which was measured by an electron spin resonance spectrometer (EMXnano, and German Bruker). The light source was 365 nm mercury lamp with 300 W power.

Irradiance Experiment

The γ-ray radiation source was cobalt-60, which was provided by Shandong Quangang Radiation Technology Co., LTD. Silicone elastomers and PNCs - Silicone elastomers were irradiated up to 300 KGy in air (dose rate was 50 Gy/min).

Computational details

All the calculations are performed in the framework of the density functional theory (DFT) with the projector augmented plane-wave method, as implemented in the Vienna ab initio simulation package. The generalized gradient approximation proposed by Perdew-Burke-Ernzerhof (PBE) is selected for the exchange-correlation potential. The cut-off energy for plane wave is set to 500 eV. The energy criterion is set to 10^{-5} eV in the iterative solution of the Kohn-Sham equation. All the structures are relaxed until the residual forces on the atoms have declined to less than 0.02 eV/Å. To avoid interlaminar interactions, a vacuum spacing of 20 Å is applied perpendicular to the slab.

The adsorption energy Eads is expressed as

$$E_{ads} = E_{total} - E_{PNCs} - E_{free\ radical}$$

where $\mathrm{E_{total}}$ is the total energy of slab PNCs model with free radical adsorption, $\mathrm{E_{PNCs}}$ is the energy of a PNCs slab, and $\mathrm{E_{free\ radical}}$ is that for a free radical molecule.

Table S1. Adsorption energy for different free radicals binding to PNCs.

	slab model	Total energy (eV)	Basal energy (eV)	molecular energy (eV)	adsorption energy (eV)
1	$CsPbBr_3$-Pb-H	-243.15	-241.69	-0.03	-1.43
2	$CsPbBr_3$-Br-H	-243.03	-241.69	-0.03	-1.30
3	$CsPbBr_3$-Pb-$SiOCH_3$	-314.45	-241.69	-71.65	-1.11
4	$CsPbBr_3$-Br-$SiOCH_3$	-313.71	-241.69	-71.65	-0.37
5	$CsPbBr_3$-Pb-CH_2SiOCH_3	-330.52	-241.69	-88.00	-0.83
6	$CsPbBr_3$-Br-CH_2SiOCH_3	-330.00	-241.69	-88.00	-0.31

Table S2. Pb-Br bond length for different free radicals binding to PNCs-Pb.

	slab model	$Pb\text{-}Br_1$(Å)	$Pb\text{-}Br_2$(Å)	$Pb\text{-}Br_3$(Å)	$Pb\text{-}Br_4$(Å)
	$CsPbBr_3$	3.02	3.02	3.03	3.02
1	$CsPbBr_3$-Pb-H	3.03	3.00	2.96	2.99
2	$CsPbBr_3$-Pb-$SiOCH_3$	2.98	2.91	3.05	3.14
3	$CsPbBr_3$-Pb-CH_2SiOCH_3	3.07	2.99	2.97	3.05

Table S3. Br-Pb bond length for different free radicals binding to PNCs-Br.

	**slab model**	**Br-Pb$_1$ (Å)**	**Br-Pb$_2$ (Å)**
	$CsPbBr_3$	3.02	3.02
1	$CsPbBr_3$-Br-H	3.34	3.32
2	$CsPbBr_3$-Br-$SiOCH_3$	3.18	2.92
3	$CsPbBr_3$-Br-CH_2SiOCH_3	2.97	3.07

Table S4. Bader charge for different free radicals binding to PNCs.

No.	slab model	Valence electrons	Total charge	Bader	description
1	$CsPbBr_3$-Pb-H	1	1.2444	-0.2444	H gets 0.2444 e
2	$CsPbBr_3$-Br-H	1	0.8876	0.1124	H loses 0.1124 e
3	$CsPbBr_3$-Pb-$SiOCH_3$	31	30.7879	0.2121	$SiOCH_3$ loses 0.2121 e
4	$CsPbBr_3$-Br-$SiOCH_3$	31	30.9793	0.0207	$SiOCH_3$ loses 0.0207 e
5	$CsPbBr_3$-Pb-CH_2SiOCH_3	37	36.9938	0.0062	CH_2-$SiOCH_3$ loses 0.0062 e
6	$CsPbBr_3$-Br-CH_2SiOCH_3	37	37.0128	-0.0128	CH_2-$SiOCH_3$ gets 0.0128 e

Table S5. PNCs- silicone elastomers with the addition of different mass fractions of PNCs

	**Before irradiation**		**After irradiation**	
Mass fraction (%)	Stress (MPa)	Strain (%)	Stress (MPa)	Strain (%)
**0**	**3.35**	**381**	**1.43**	**75**
**0.69**	3.36	377	2.67	156
**1.38**	3.38	325	2.94	162
**2.75**	**3.29**	**366**	**2.94**	**184**
**4.13**	3.22	365	2.89	154
**6.88**	3.30	365	2.92	174

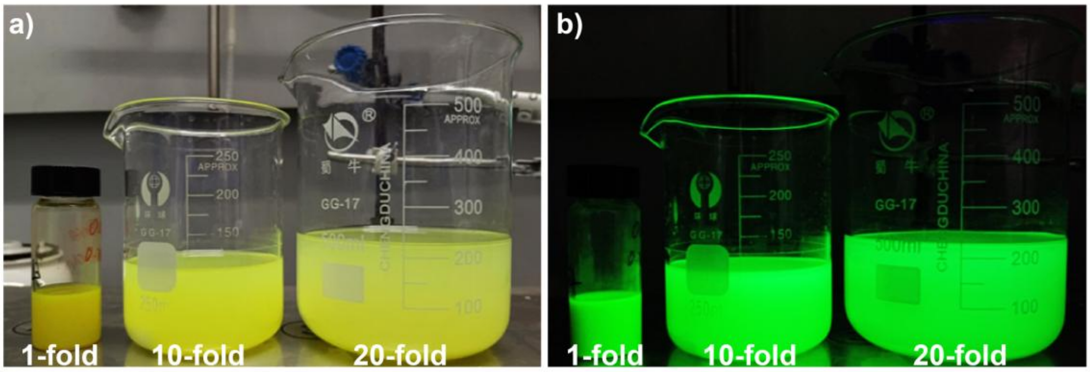

Figure S1. Photographs of different scaled-up folds of $CsPbBr_3$ PNCs crude solution.

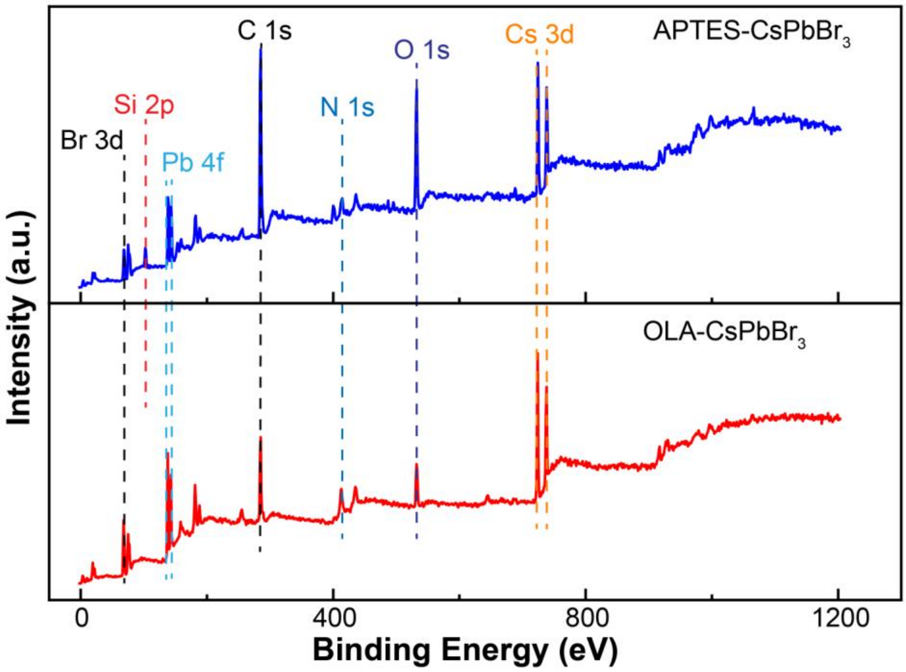

Figure S2. XPS spectra of APTES-PNCs and OLA-PNCs, marked with all the elements contained in the samples. The peaks of elemental Si were obviously observed for APTES-PNCs whereas not for OLA-PNCs.

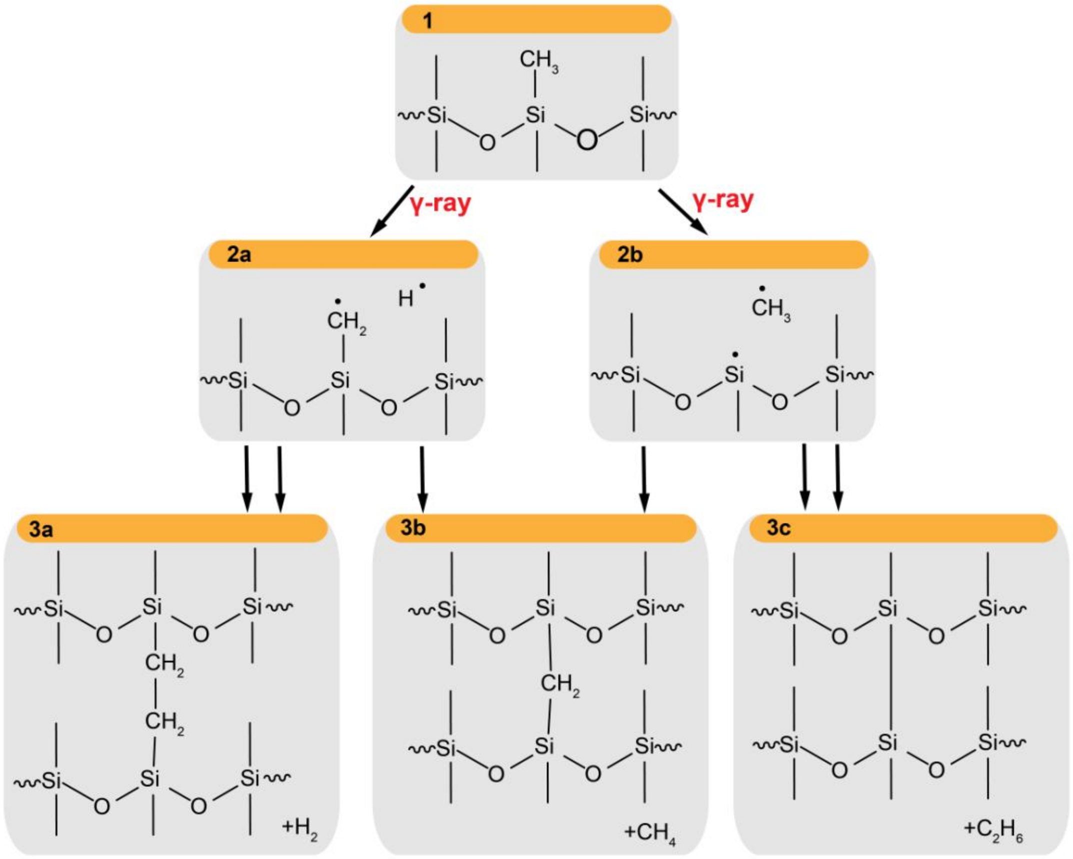

Figure S3. Schematic mechanism of the destruction of the cross-linked structure of PDMS irradiated by γ-rays. The first step was the formation of free radical pairs, and these free radicals would react with the main chain or other free radicals in a random manner.

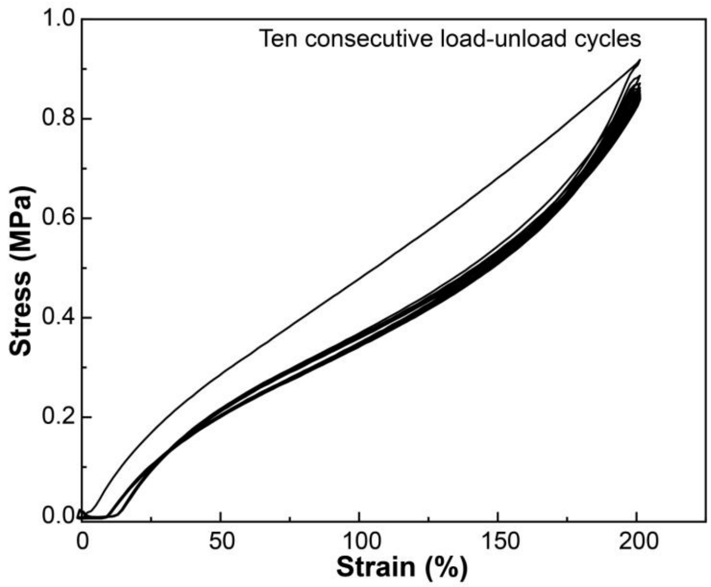

Figure S4. 10-cycle loading–unloading test of silicone elastomer at 200% strain.

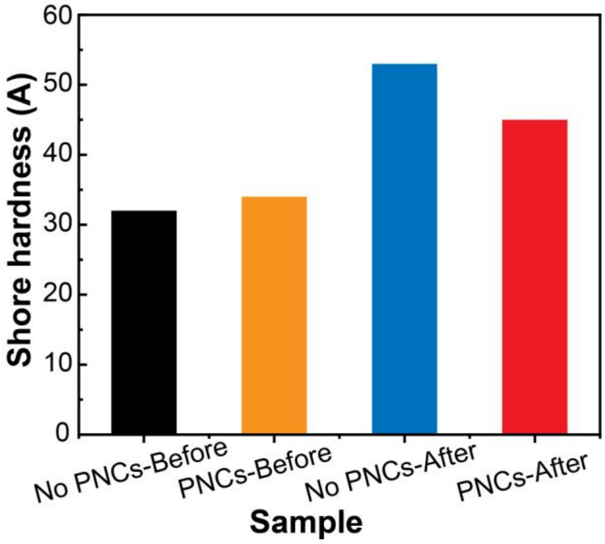

Figure S5. Shore hardness A of silicone elastomer and PNCs-silicone elastomer before and after irradiation.

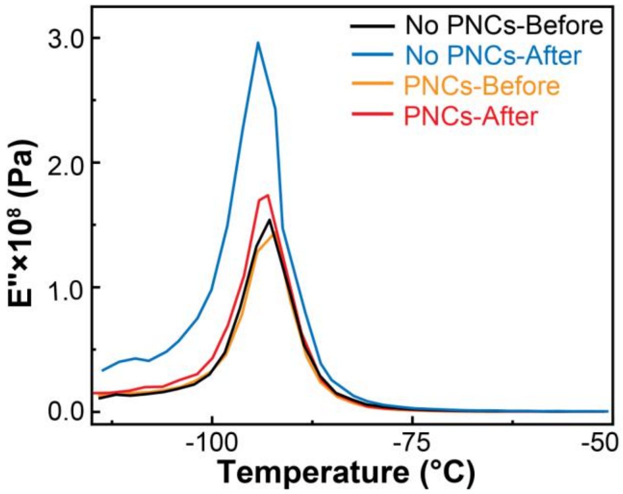

Figure S6. Loss modulus of silicone elastomer and PNCs-silicone elastomer before and after irradiation.

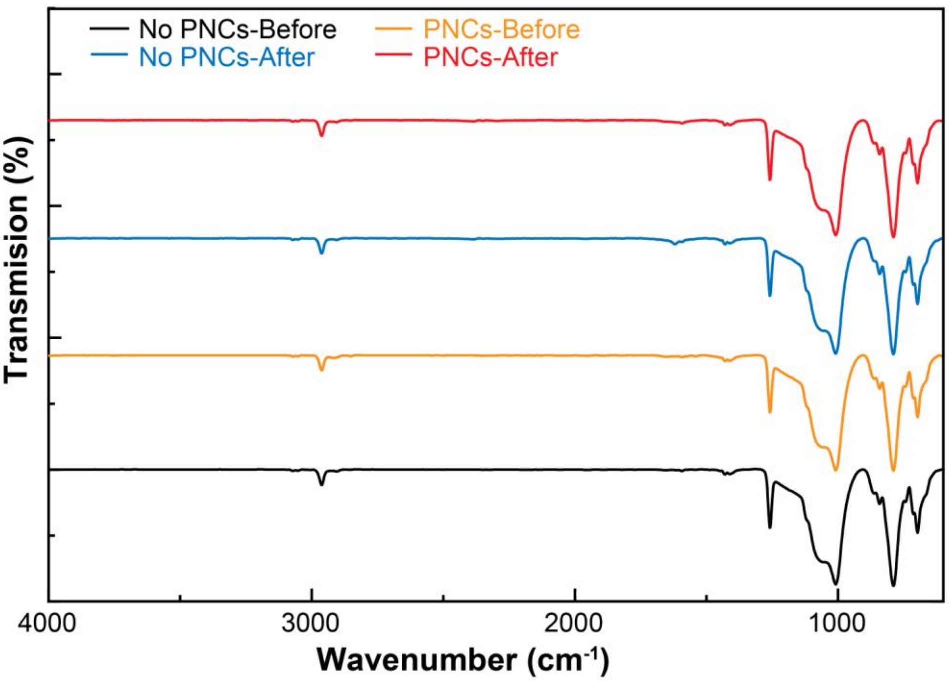

Figure S7. FT-IR spectra of silicone elastomer and PNCs-silicone elastomer before and after irradiation.
